# Valorization of Olive By-Products: Innovative Strategies for Their Production, Treatment and Characterization

**DOI:** 10.3390/foods11060768

**Published:** 2022-03-08

**Authors:** Cosima D. Calvano, Antonia Tamborrino

**Affiliations:** 1 Inter-Department Center SMART, Department of Chemistry, University of Bari Aldo Moro, Via Orabona 4, 70126 Bari, Italy; 2 Department of Agricultural and Environmental Science, University of Bari Aldo Moro, Via Amendola 165/A, 70126 Bari, Italy

Presently, olive oil production signifies a valuable economic income for Mediterranean countries, where approximately 98% of the world’s production is established [1]. The cultivation of olive trees and the production of olive oil generate massive amounts of solid wastes, including olive tree pruning (OTP), olive leaves (OL), olive stone (OS) and liquid effluents such as olive pomace (OP), olive oil mill wastewaters (OOMW) and olive mill waste (OMW) [2] depending on the techniques used for oil manufacture [3,4,5,6,7]. Olive by-products have long been considered as a challenging waste material impacting environmental protection, but this view has recently been reformed, leading to their recognition as a source of high-added value compounds as phenols and antioxidants that can be re-used as natural additives in the food, cosmetic and drug industries. This Special Issue (SI) entitled “Valorization of Olive By-Products: Innovative Strategies for Their Production, Treatment and Characterization” focuses both on the recent advancements in technologies for the extraction of target compounds from olive by-products and on their characterization by advanced analytical techniques with the aim of valorizing these compounds. This SI consisted of one review and eight research articles that substantially contributed to the mission of *Foods*, i.e., to growth scientists’ and readers’ information on food science, including food engineering and technology, food quality and safety, food analytical methods, nutraceuticals and functional foods.

The review [8] offers first an overview of the general characteristics in terms of antioxidant, anti-inflammatory, anti-atherogenic, anti-cancer, and antimicrobial activities and the main applications of OL, OP and OMW by-products with a special emphasis on the correlation between the content of high-added value compounds and their reuse; in the second part, this review focuses on the literature concerning the use of mass spectrometry (MS) techniques for their deep characterization in these matrices. The analytical methods useful for providing detailed structural information and for quantifying bioactive compounds in olive oil by-products are based on the coupling between GC or liquid chromatography (LC) and MS. The soft ionization sources based on electrospray (ESI), atmospheric pressure chemical (APCI) [9] and matrix-assisted laser desorption (MALDI) [10] have become versatile configurations for identifying phenolic-related compounds in waste. For instance, the use of high resolution/accuracy MS has opened interesting perspectives for an even deeper structural characterization of bioactive phenolic compounds in each part of the olive plant. It becomes clear that the chemical composition of olive by-products can be quite dissimilar from that of olive fruit, olive drupes and olive oils [11] and a high degree of complexity in terms of isomeric forms are recognized [12,13,14]. Further, it is stressed as the distribution and integrity of phenolic compounds are generally affected by various parameters, including olive cultivar and geographical origin [15], ripening stage at the harvesting time [16], agronomic and technological practices [17,18], and storage conditions [19,20]. Interestingly, available quantitative data of specific compounds are also reported and discussed in the perspective of individuating which compounds or class of compounds may deserve the development of appropriate recovery strategies from olive oil production by-products.

For example, one of the papers of this SI [21] reports on the analytical characterization of four diverse pools of biomass by-products such as OTP, OL, OS and extracted olive pomace (EOP) to highlight their potential recovery of the water-extractable fraction within a biorefinery context. The final aim was to assess the occurrence of high-value-added compounds as carbohydrates, proteins, alditols and to ascertain a comparison between the four different olive biomasses of their structural composition and quantity in the water extractives. These four by-products had definite compositions depending on the part of the plant they come from and the level of processing they have encountered. Thus, OTP, containing thin branches, olive wood and olive leaves, had a much higher structural carbohydrates content (about 35%) than OL alone, which only had 19% of the total dry weight. EOP, containing mainly the rest of the olive fruit, presented a low content in cellulose (11%) and hemicellulose (12%), while OS showed the highest value among the four substrates of cellulose and hemicellulose, close to 50%. This rather high carbohydrate content in OS and OTP could suggest a good potential for the utilization of these fractions as feedstocks in a biorefinery context. Among the four olive by-products, OS reported the highest organic acid content (16% of the total aqueous extractives weight) equally divided between acetic, coming mainly from the hydrolysis of acetyl groups in hemicellulose and other acids. A significant concentration of acetic and other aliphatic acids in OS could indicate a good potential for the recovery of these products from hemicellulose.

Cedola et al. [22] illustrate the valorization of leaves by-products as a food ingredient in a cereal-based food popular all around the world as “taralli”. Due to the promising antimicrobial and antioxidant action of phenols present in olive by-products, many researchers try to integrate by-products in the form of powder or extract in food; here, the extract obtained by ultrasound-assisted procedure from dried olive leaves was used as an ingredient instead of white wine [22]. From the sensory point of view, the enriched product was found generally acceptable as the control sample; the enriched “taralli” showed lower friability than the conventional product, but it still recorded an overall quality score well above the acceptability limit (score = 5). The total content of phenols, flavonoids and antioxidant capacity were measured on both cooked and uncooked samples. The obtained results indicated that olive leaves extract improves the nutritional quality of enriched food, going from 0.53 mg GAE/g dw to 0.72 mg GAE/g dw for the uncooked sample, and from 0.43 mg GAE/g dw to 0.61 mg GAE/g dw for the cooked one suggesting that cooking process did not affect the concentration of polyphenols, flavonoids and antioxidant activity. For a nutritional assessment, it is also necessary to consider the bio-accessibility of nutrients so to confirm the release of the compounds after digestion, as they can undergo changes or even degradation in the gastrointestinal digestion process. High-Performance Liquid Chromatography with Diode-Array Detection (HPLC-DAD) of the three most abundant phenols, oleuropein, hydroxytyrosol and verbascoside, was carried out at the three stages of the digestion process (oral, gastric and intestinal). Oleuropein reached the duodenum, but the intestinal phase reduced its quantity; hydroxytyrosol and verbascoside, even if much lower than the oleuropein value, presented a trend quite similar from the middle to the end of the digestion process. These in vitro results suggest as micro- or nanoencapsulation of phenolic compounds could be an efficient technique to prevent degradation and protect the enriched food against environmental and gastrointestinal system conditions. In this SI, this aspect has been included too, describing the characterization, antioxidant activity and oxidative stability of OP extract encapsulated in rocket seed gum (RSG) and chia seed gum (CSG) nanoparticles used as wall material [23]. The proposed nanoparticles (NPs) were characterized by using particle size and zeta potential measurement, differential scanning calorimeter, Fourier-transform infrared spectroscopy, scanning electron microscope, encapsulation efficiency, in vitro release, and antioxidant activity analyses. The phenolic profiles and physicochemical properties of OP, RSG and CSG were also determined. Further, NPs were used to form oil in water emulsions, later evaluated by oxitest. In vitro release study showed that the encapsulation of OP extract (OPE) in RSGNPs and CSGNPs led to a deferral of the OPE released at physiological pH. The total phenolic content and antioxidant capacity of OPE-loaded RSGNPs and CSGNPs was higher compared to blank RSGNPs and CSGNPs. These results indicate that both RSG and CSG could be successfully applied as wall material for cold-pressed olive pomace nanoencapsulation to prevent the degradation of main phenolic compounds. On the other hand, the application of green techniques to extract bioactive compounds from olive leaves or other by-products could be of great interest, not only for the attainment of these natural active compounds but also for the possibility of re-using significant by-products from the industry. For example, a few recent reports have studied the influence of the different extraction conditions on the achievement of phenolic antioxidants from these substances. López-Salas et al. [24] have conducted research on preliminary investigation of different drying systems to preserve hydroxytyrosol and its derivatives in olive oil filter cake pressurized liquid extracts. This aspect appears interesting considering that the filtration process of EVOO is widely applied in most olive-oil industries of the main producing countries of the Mediterranean as a final step prior to selling the oil. In this sense, filter cakes are generated in large amounts in the final step of cloudy oil filtration. The main objective of the filtration process is to remove humidity and suspended solids of EVOO to obtain good quality oil for consumer acceptance, as well as to improve oil stability. The loss of water leads to a decrease in polar compounds content in EVOO, including phenolic compounds. This fact originates filter cakes enriched in these compounds of interest with many potentials, such as hydroxytyrosol and derivatives. In this line, the potential combination of an advanced extraction technique (pressurized liquid extraction, PLE) coupled to vacuum-, freeze- and spray-drying systems to obtain the best innovative process to recover hydroxytyrosol and its antioxidant derivatives from filter cake generated during EVOO production was evaluated.

A marked influence of the drying process on the composition of these extracts was noticed. Therefore, the PLE extracts submitted to freeze-drying showed a higher content of phenolic compounds compared to other drying techniques. Regarding this study, the quantified amounts of total phenolic alcohols varied from 10,364 to 24,066 mg/kg of the extract, depending on the drying technique, whereas the content of total secoiridoids was from 2202 to 4654 mg/kg of dried extract.

Among olive by-products, the vegetation waters contain many phenols, which have several health-beneficial properties. Precisely because of their antioxidant power combined with antibacterial activity, they are used to improve the conservation and quality of meat and fruit. A well-articulated study presented in this SI highlights the use of a phenolic extract from vegetation waters on the shelf-life of deep-water rose shrimp [25] and on the quality and sensory profile of cooked pink shrimps treated with an olive vegetation water phenolic extract [26]. Preserving the foodstuffs’ quality during storage without adding synthetic additives is a strategy that the fish industry has been trying to pursue in recent years. Melanosis and microbiological deterioration are common problems during the post-harvest storage of crustaceans, damaging their sensory features and decreasing their quality, shelf-life and subsequently, their commercial value.

The olive vegetation water phenolic extract, as a natural additive tested in this study on pink shrimp [25], showed several interesting implications. It was able to delay the alterative phenomena by slowing down lipid oxidation, microbial development and the volatile nitrogen compounds’ formation in a manner proportionally active to the dose of extract used. The effects on melanosis formation were less evident. Phenolic compounds alone, at a concentration of 2 g/L, showed a marginal, yet not significant (*p* < 0.05), inhibitory effects on the black spots’ progression. However, the treatment with 1 g/L of phenols, added to a 0.25% sodium metabisulfite solution, was able to delay melanosis with an efficacy equal to sodium metabisulfite solution alone at a concentration of 0.5%, which is normally used by the manufacturers for anti-melanosis in shrimp. This result is very promising, given the well-known adverse reactions associated with sulfites additives. Indeed, being able to treat foods with lower quantities of sulfites allows one to obtain a healthier product and, on the other hand, a product acceptable to the consumer. In this regard, several experimental trials have also been conducted aimed at reaching more stability in the phenolic extract derived from olive vegetation water over time [25].

Miraglia et al. [26] have presented the result regarding the quality traits and sensory profile of cooked pink shrimps treated with an olive vegetation water phenolic extract. In order to evaluate significant sensory differences among the samples at each phase of storage, a discriminant sensory test was carried out on the cooked shrimps. The quantitative-descriptive analysis (QDA) method (UNI EN ISO 13299:2016) was used, and an unstructured, linear graphical scale (100 mm) was converted to numerical values (0 to 10 conventional units). Sensory quality was characterized based on sensory attributes, grouped in the following: appearance, odor, taste, texture and overall quality. Interpretation of the sensory test using an exploratory PCA model and correlation analyses revealed significant improvements conferred by the extract to the microbiological profile and consequently to the sensory characteristics during storage. The bactericidal and antioxidant activity of the phenolic compounds was proportional to their concentration, as were the pleasant sensory attributes. In this regard, the phenolic fractions found in the shrimps after cooking, albeit at different percentages of reduction, enriched the nutraceutical value of the rose shrimps, which in addition to being safer with longer-lasting shrimp sensory characteristics, were also without or with less use of synthetic additives. Furthermore, at the concentrations used by the authors, the phenolic extract did not modify either the sensory characteristics of the fresh shrimp or during the storage period. Finally, sulfites combined with the extract demonstrated a less effective antibacterial action, although they appear to have protected the phenolic compounds from the oxidative processes of cooking and storage.

According to today’s need for an ecological transition, the waste of oil mill by-products may be introduced in a circular-economy-based supply chain. Energetic characteristics, combustion behavior, and densification properties made this biomass feedstock useable for energy production through different processes. The vegetation water (OMWW) and the pomace (OMSR) have an acidic pH, high values of chemical demand (COD) and biochemistry (BOD5) of oxygen, low nitrogen content and the presence of lipids and a phenolic fraction. The high organic load represents a significant energy potential and would make the olive mill by-products one of the most suitable agro-industrial wastes for anaerobic digestion [27].

In Tamborrino et al. [28], an anaerobic digestion plant has been studied; it is one of the first full-scale plants which uses only olive oil by-products. Recent studies have experimented at a laboratory scale on the possibility of producing biogas through the anaerobic digestion of 100% of olive oil waste. The aim was to analyze the real-scale process of anaerobic digestion of by-products of olive oil production to evaluate the feasibility, process variables, design and operational parameters for this specific anaerobic digestion industrial plant starting with olive oil by-products. The biomass considered for the experimental tests was essentially pulp and pitted olive pomace. Pitted Olive pomace is a by-product consisting of the shell and pulp remaining after olive oil extraction, without the kernel that is usually removed from the virgin pomace immediately downstream of its production in the mill.

The results of the experimentation show the possibility of feeding an industrial anaerobic digestion plant exclusively with olive oil effluents, and the performance of the studied plant can be considered comparable with the data of other full-scale plants fed with biomass made up of other types of generally tested organic matrices. The results show very appreciable specific biogas production with a high percentage of methane. The produced biogas had a methane percentage of about 60%, and the specific production (over total volatile solids, TVS) of methane was of the order of 0.70 m^3^ methane/kgTVS. The chemico-physical analyses show optimal values of the process stability indexes and of the parameters that define the regularity of the kinetics of anaerobic digestion.

The use of self-produced electric energy and the sale of the excess rate to the network manager should not be considered the only factor of return. Instead, it should be considered that the energy recovery of olive oil by-products also allows a significant reduction of their disposal costs, the possibility of selling the pits, as well as the digestate as a quality improver. Moreover, the mill can take advantage of the benefit of the environmental commitment in primary production that, with the same quality, would allow the selling prices of produced olive oil to rise even more.

Regarding this prospective research, composting is a simple method to use, has few process requirements, has relatively low investment costs, and can be conveniently carried out using organic waste matrices from different agro-food chains. Considering the importance of valorizing organic waste from the food industry, minimizing the environmental impact of the sewage sludge, and, finally, the need to return mature organic substance to the soil as a soil improver for crops, a research paper of the SI [29] had the purpose of evaluating the technical feasibility of co-composting of four types of organic wastes, one of this represented by the olive mill pomace. In particular, the research has aimed to set up a composting plant equipped with a machine for turning and shredding the windrow. To conduct this research, two piles (A and B) that differ in the C/N ratio were prepared using the following matrices: pitted olive mill pomace (OMP), sewage sludge from vegetable processing (SS), fresh residues from artichoke processing residues (AR), and wheat straw (WS). During a two-year period, an evaluation was carried out of the effects of two different obtained types of compost on the main qualitative parameters of processing tomato (cv Ulisse S&G Syngenta Seeds S.p.A.) and durum wheat crops (cv Saragolla S&G Syngenta Seeds S.p.A.). The results of this study showed that a part of the required fertilizing elements of the tomato and wheat crops could be provided by compost and, thus, alleviate the environmental hazards on agroecosystems (e.g., fertilizer leaching). This study has shown that it is possible to carry out a composting process of different organic matrices from different food chains carefully mixed by adequately controlling/adjusting the process parameters until a stable compost for agronomic purposes is obtained.
